# Oxidant Stress and Signal Transduction in the Nervous System with the PI 3-K, Akt, and mTOR Cascade

**DOI:** 10.3390/ijms131113830

**Published:** 2012-10-26

**Authors:** Kenneth Maiese, Zhao Zhong Chong, Shaohui Wang, Yan Chen Shang

**Affiliations:** 1 Laboratory of Cellular and Molecular Signaling, Newark, NJ 07101, USA; E-Mails: zzchong@yahoo.com (Z.Z.C.); wsh2078@gmail.com (S.W.); ycshang2000@yahoo.com (Y.C.S.); 2 Cancer Institute of New Jersey, New Brunswick, NJ 08903, USA; 3 New Jersey Health Sciences University, 205 South Orange Avenue, Newark, NJ 07101, USA

**Keywords:** Akt, Alzheimer’s disease, apoptosis, autophagy, diabetes mellitus, Huntington’s disease, mammalian target of rapamycin (mTOR), oxidative stress, Parkinson’s disease, phosphoinositide 3-kinase (PI 3-K), SIRT1

## Abstract

Oxidative stress impacts multiple systems of the body and can lead to some of the most devastating consequences in the nervous system especially during aging. Both acute and chronic neurodegenerative disorders such as diabetes mellitus, cerebral ischemia, trauma, Alzheimer’s disease, Parkinson’s disease, Huntington’s disease, and tuberous sclerosis through programmed cell death pathways of apoptosis and autophagy can be the result of oxidant stress. Novel therapeutic avenues that focus upon the phosphoinositide 3-kinase (PI 3-K), Akt (protein kinase B), and the mammalian target of rapamycin (mTOR) cascade and related pathways offer exciting prospects to address the onset and potential reversal of neurodegenerative disorders. Effective clinical translation of these pathways into robust therapeutic strategies requires intimate knowledge of the complexity of these pathways and the ability of this cascade to influence biological outcome that can vary among disorders of the nervous system.

## 1. Introduction

Oxidative stress through the generation of reactive oxygen species (ROS) can severely impact the nervous system and related pathways that control immune function and cellular metabolism during aging. ROS are formed through superoxide free radicals, hydrogen peroxide, singlet oxygen, nitric oxide (NO), and peroxynitrite [1–3]. ROS can result in mitochondrial dysfunction, the loss of DNA integrity, and the misfolding of proteins that promote cell injury in the nervous system [2,4,5]. Antioxidant systems in the body that include catalase, superoxide dismutase, glutathione peroxidase, and vitamins C, D, E, and K limit the production of ROS to non-toxic levels [6–13]. However, excessive production of ROS or impairments in the endogenous antioxidant system can lead to oxidative stress and cell death through DNA degradation, mitochondrial dysfunction, and protein misfolding [2,3,14–17]. Oxidative stress can lead to cognitive loss [14,18–20], Alzheimer’s disease (AD) [1,13,20–24], Huntington’s disease (HD) [25–31], Parkinson’s disease [1,9,26,32–39], epilepsy [25,26,28,40–43], and acute injury such as during stroke and trauma [44–54]. Oxidative stress also modulates inflammatory cell function [55–60] and alters cellular metabolism that leads to complications of diabetes mellitus [5,61–71]. Although multiple cellular pathways are impacted during oxidative stress in the nervous system, the kinase cascade that involves phosphoinositide 3–kinase (PI 3-K), protein kinase B (Akt), and the mammalian target of rapamycin (mTOR) offers exciting prospects for the development of new treatment strategies since these pathways are closely integrated and directly affect both acute and chronic neurological disorders [25,55,72–74]. Greater understanding of these pathways can foster novel as well as safe clinical treatment avenues for disorders of the nervous system.

## 2. Oxidant Stress and Cellular Injury

### 2.1. Cellular Survival and Demise during Neurodegeneration

**Figure 1:**
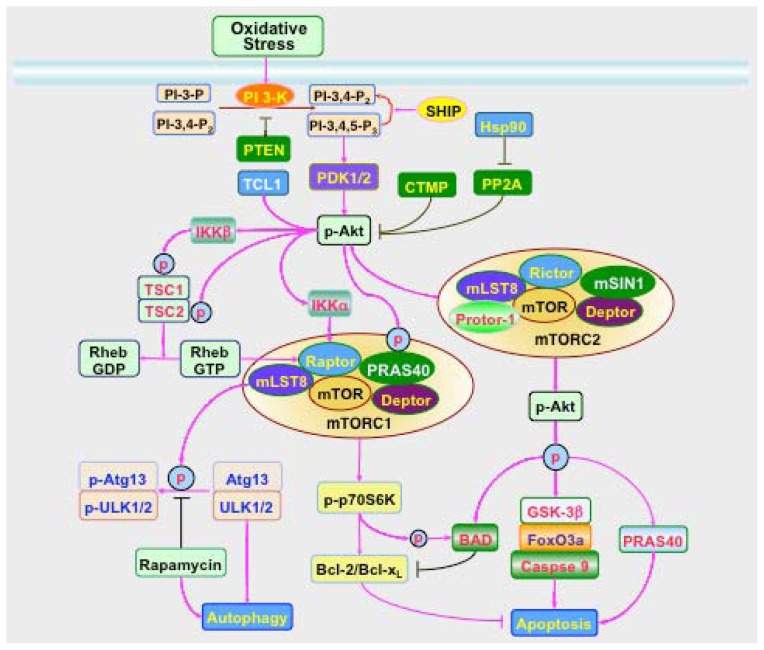
Signal transduction pathways of the PI 3-K, Akt, and mTOR cascade. During oxidative stress, multiple pathways are affected that involve PI 3-K, Akt, and mTOR that ultimately interface with programmed cell death pathways of apoptosis and autophagy. Activation of phosphoinositide 3 kinase (PI 3-K), such as by tropic factors that include erythropoietin can promote the production of phosphatidylinositide (3,4)-biphosphate (PI-3,4-P_2_) and phosphatidylinositide (3,4,5)-triphosphate (PI-3,4,5-P_3_) that recruits Akt to the plasma membrane. This recruitment activates phosphoinositide dependent kinase 1 (PDK1) and PDK2, leading to Akt phosphorylation. Akt activity can be blocked by the phosphatase and tensin homolog deleted from chromosome 10 (PTEN), SH2 domain-containing inositol phosphatase (SHIP), and carboxyl-terminal modulator protein (CTMP). Akt activity can be enhanced by the T cell leukemia/lymphoma 1 (TCL1) and 90 kDa heat shock protein (Hsp90) that can inhibit protein phosphatase 2A (PP2A). Akt can activate mTORC1 through phosphorylating TSC2 and disrupting the interaction between TSC2 and TSC1. Akt may also activate mTORC1 through I-kappaB kinase (IKK). IKKα associates with Raptor and IKKβ that can phosphorylate TSC1 and suppress TSC1 and its interaction with TSC2. In addition, Akt can directly phosphorylate proline rich Akt substrate 40 kDa (PRAS40) to reduce PRAS40 binding to regulatory associated protein of mTOR (Raptor) and thereby activate mTORC1. Upon activation, mTORC1 phosphorylates its downstream targets p70 ribosome S6 kinase (p70S6K) to phosphorylate pro-apoptotic protein BAD and increase the expression of Bcl-2/Bcl-x_L_ which functions as an anti-apoptotic protein. mTORC1 activation also inhibits autophagic proteins autophagy related gene 13 (Atg13) and UNC-51 like kinase 1/2(ULK1/2) through phosphorylation to prevent autophagy. Rapamycin, an inhibitor of mTOR, can prevent this process and foster autophagy. mTOR signaling inhibits apoptosis though activation of Akt that inhibits “pro-apoptotic” proteins FoxO3a, glycogen synthase-3β (GSK-3β), BAD, and PRAS40.

Oxidative stress pathways that involve the PI 3-K, Akt, and mTOR cascade can lead to cellular injury through the processes of apoptosis and autophagy [39,75] (Figure 1). During neurodegenerative disorders, apoptosis may be a significant contributor of cell dysfunction and loss. Apoptotic DNA fragmentation [76] and caspase activation is present [77] in the brains of patients with Alzheimer’s disease (AD). Alzheimer’s disease models have demonstrated apoptotic proteins in the brain [78]. Apoptotic neuronal nuclei and caspase 3 have been identified in the postmortem nigra of Parkinson’s disease (PD) patients [79]. For immune mediated cells of the brain, loss of microglia can occur through apoptotic cell death [80,81]. In regards to autophagy, this process may play a dual role that can either protect cell survival or potentiate cell injury. During oxidative stress, autophagy can lead to cell death in cerebral astrocytes [82], in cortical neurons [83], in spinal cord motor neurons [84], in purkinje neurons [85], and in sympathetic neurons [86]. Other toxins such as glutamate, potassium deprivation, and staurosporine also lead to autophagy and cell death [85]. During metabolic disease, apoptosis can lead to vascular complications, impair endothelial cell survival, destroy immune mediated cells, inhibit wound repair, and injure neurons [1,87,88]. However, autophagy and apoptosis may have similar outcomes that are intertwined. The agent methamphatamine leads to cell death through both apoptosis and autophagy by inhibiting the disassociation of the “apoptotic-autophagic complex” Bcl-2/Beclin 1 complex [89]. Autophagy and apoptosis also may have opposing roles. Apoptosis may conversely require the inhibition of autophagy [90] or apoptotic neuronal cell death may be independent of the onset of autophagy [91]. Under some conditions, autophagy can offer cytoprotection during neurodegenerative disorders [92,93]. Autophagy may be necessary to protect against neuronal cell loss and α-synuclein toxicity in PD [92]. Mutation of α-synuclein and accumulation of wild-type α-synuclein in dopaminergic neurons have been associated with progression of Parkinson’s disease [94]. Autophagy is necessary for the clearance of aberrant α-synuclein in neurons since inhibition of this autophagic pathway leads to accumulation of high molecular weight and detergent insoluble α-synuclein and neurotoxicity [94]. Mutant α-synuclein, which is poorly internalized into lysosomes, also is degraded by macroautophagy [92]. During metabolic disease such as diabetes mellitus (DM), autophagy may be necessary to remove misfolded proteins and eliminate non-functioning mitochondria in β-cells to prevent β-cell dysfunction and the onset of DM [95]. Exercise in mice has been shown to initiate autophagy and regulate glucose homeostasis [96]. However, the generation of advanced glycation end products (AGEs) during DM also may lead to autophagy that contributes to vascular smooth muscle proliferation, atherosclerosis [97], and potential cardiomyopathy [64]. The elevation of free fatty acids in cell models of DM suggest that fatty acids may be necessary to activate autophagy in beta cells through endoplasmic reticulum stress [98].

### 2.2. Apoptotic Early and Late Phases

Apoptosis consists of two distinct components that involve genomic DNA degradation and the loss of plasma membrane lipid asymmetry [2]. The loss of asymmetry of membrane phosphatidylserine (PS) distribution is an early component of apoptosis that can be reversible [99]. A later event, the cleavage of genomic DNA into fragments can occur once a cell has been committed to die [100]. Once initiated, genomic DNA degradation usually is not reversible. Enzymes responsible for DNA degradation include the acidic cation independent endonuclease (DNase II), cyclophilins, and the 97 kDa magnesium-dependent endonuclease. Three separate endonuclease activities exist in neurons that include a constitutive acidic cation-independent endonuclease, a constitutive calcium/magnesium-dependent endonuclease, and an inducible magnesium dependent endonuclease [22]. Both membrane PS exposure and genomic DNA degradation are considered to be the outcomes of a series of activation of nucleases and proteases that occurs late during apoptosis [1,88]. Exposure of membrane PS residues during oxidative stress can occur with sepsis, ischemia, vascular clot formation, and β-amyloid deposition [101–103]. The early phase of apoptosis can tag cells with membrane PS residues to alert inflammatory cells to engulf and remove injured cells [104,105]. For this to occur such as during periods of oxidative stress, inflammatory cells increase their expression of the membrane phosphatidylserine receptor (PSR) [106–108]. To promote cell survival, modulation of inflammatory cell activation is necessary, since removal of temporarily injured cells expressing membrane PS residues can lead to the loss of functional cells [109,110].

### 2.3. The Sub-Constructs of Autophagy

In contrast to apoptosis, autophagy has three different categories known as microautophagy, macroautophagy, and chaperone-mediated autophagy [99,111,112]. Macroautophagy consists of the degradation of cytoplasmic material and the sequestration of the cytoplasmic protein and organelles into autophagosomes. Autophagosomes fuse with lysosomes for degradation and are then recycled for future cellular processes [113]. Microautophagy represents the sequestration of cytoplasmic components by invagination of the lysosomal membrane. Vesicles subsequently formed are transferred to the lumen of the lysosomes for digestion. In chaperone-mediated autophagy, the cytoplasmic component is transported by cytosolic chaperones to the receptors on the lysosomal membranes for translocation across lysosomal membranes into the lumen.

In yeast, thirty-three autophagic related genes (*Atg*) have been identified. Of this family of genes, Atg1 and Atg13 are associated with the PI 3-K, Akt, and mTOR cascade [114–116]. In yeast, Atg13 (also known as Apg13) is phosphorylated through a Tor dependent mechanism, leading its release from Atg1 and a reduction in Atg1 activity. The Tor kinase was first isolated in *Saccharomyces cerevisiae* through the analysis of rapamycin toxicity using rapamycin-resistant TOR mutants in yeast that resulted in the identification of the yeast genes *TOR1* and *TOR2* that encode two isoforms in yeast Tor1 and Tor2 [117]. In mammals, a single gene *FRAP1* encodes mTOR [99,118]. Upon starvation or Tor inhibition, Atg13 is dephosphorylated, binds to, and activates Atg1, leading to autophagosome formation [116]. In mammals, a similar regulation of autophagy through mTOR also exists. Two mammalian homologues of Atg1, UNC-51 like kinase 1 (ULK1) and ULK2, have been identified [119–121]. Mammalian Atg13 binds to ULK1, ULK2, and FIP200 (FAK-family interacting protein of 200 kDa) to activate ULKs and facilitate the phosphorylation of FIP200 by ULKs [122,123]. Similar to TOR in yeast, mTOR phosphorylates the mammalian homologue Atg13 and the mammalian Atg1 homologues ULK1 and ULK2 to block autophagy [123]. The focal adhesion kinase family interacting protein of 200 kDa (FIP200) has been identified as a ULK binding protein. FIP200 and Atg13 are vital for the stability and activation of ULK1. Mammalian Atg13 binds to ULK1/2 and FIP200 to activate ULKs and facilitate the phosphorylation of FIP200 by ULKs [123]. mTOR activation prevents autophagy in mammalian cells through inhibition of the ULK-Atg13–FIP200 complex by phosphorylating Atg13 and ULKs. In the absence of mTOR activity, dephosphorylation of ULKs and Atg13 ensues leading to the induction of autophagy [122,123].

## 3. Signal Transduction and Cell Survival with PI 3-K, Akt, and mTOR

### 3.1. PI 3-K and PDK

Receptor tyrosine kinase (RTK) and the G protein-coupled receptor (CPCR) are required for PI 3-K activation. Following activation, PI 3-K phosphorylates membrane lipids and mediates the transition of Akt from the cytosol to the plasma membrane by promoting the binding of Akt to PI-3,4-P_2_ and PI-3,4,5-P_3_ through the plectrin homology (PH) domain (Figure 1). Akt is then phosphorylated on the residues of serine^473^ and threonine^308^ by phosphoinositide dependent kinase (PDK) PDK1 and PDK2. PDK1 is responsible for phosphorylating Akt at threonine^308^[124]. PDK1 contains a *C*-terminal PH domain that PI-3, 4, 5-P_3_ can recruit PDK1 to the cell membrane and bind to the *C*-terminal HM domain of Akt. PDK1 cannot directly phosphorylate Akt on serine^473^, but phosphorylation of Akt on serine^473^ is necessary for the full activation of Akt. PDK2 like kinases, such as integrin-linked kinase, DNA dependent protein kinase, PKCβ, and mTORC2, have been identified to promote Akt phosphorylation on serine^473^[125]. In contrast, the phosphatase and tensin homolog deleted from chromosome 10 (PTEN), which specifically dephosphorylates PI-3,4-P_2_ and PI-3,4,5-P_3_ at the D3 position can block PI 3-K signaling and inhibit Akt activation.

### 3.2. Akt

A number of pathways can influence Akt activity during oxidative stress [126–130]. The 90 kDa heat shock protein (Hsp90) that is involved in modulating oxidative stress in cells [131] can increase Akt activity through the inhibition of inhibiting protein phosphatase 2A (PP2A). In addition, the T cell leukemia/lymphoma 1 (TCL1) protein binds to the PH domain of Akt to increase Akt activity (Figure 1). In regards to down-regulation of Akt activity, the carboxyl-terminal modulator protein (CTMP) binds to the carboxyl-terminal regulatory domain of Akt1 at the plasma membrane to prevent Akt1 from phosphorylation. The src homology 2 (SH2) domain-containing inositol phosphatase (SHIP) is an inositol 5′ phosphatase that dephosphorylates inositides and phosphoinositides on the 5′-position [55]. Both SHIP1 and SHIP2 can negatively regulate the activity of Akt. PI-3, 4, 5-P_3_ are transformed into PI-3, 4-P_2_ that is less potent than PI-3, 4, 5-P_3_ to recruit Akt. The SH2 domains containing protein-tyrosine phosphatases SHP1 and SHP2 also modulate the activity of PI 3-K. SHP1 associates with the p85 subunit of PI 3-K to negatively regulate the activation of PI 3-K. SHP2 can be necessary for agents that promote cell differentiation to lead to the activation of PI 3-K and Akt [132].

### 3.3. mTOR

In relation to mTOR, which also is known as mechanistic target of rapamycin and FK506-binding protein 12-rapamycin complex-associated protein 1 (FRAP1), Akt is a strong stimulator of mTORC1 to lead to the activation of mTORC1 [133]. As a component of the PI 3-K related kinase family that is activated through the PI 3-K and Akt, mTOR is a 289-kDa serine/threonine protein kinase that can control transcription, cytoskeleton organization, cellular survival, and cellular metabolism [25,87,99,133–135]. mTOR signaling is dependent upon the protein complexes mTOR Complex 1 (mTORC1) or mTOR Complex 2 (mTORC2) that each contain mTOR (Figure 1). p70 ribosomal S6 kinase (p70S6K) and eukaryotic initiation factor 4E (eIF4E)-binding protein 1 (4EBP1) are downstream targets of mTORC1 [99,136]. Phosphorylation of p70S6K promotes mRNA biogenesis, translation of ribosomal proteins, and cell growth [137,138]. In contrast, phosphorylation of 4EBP1 results in its inactivation. Hypophosphorylated 4EBP1 is active and binds competitively with eukaryotic translation initiation factor 4 gamma (eIF4G) to eukaryotic translation initiation factor 4 epsilon (eIF4E) that regulate translation initiation by interacting with the 5′-mRNA cap structure. The phosphorylation of 4EBP1 by mTORC1 results in its dissociation from eIF4E allowing eIF4G to interact with eIF4E and promotes protein translation [139,140]. Tuberous sclerosis complex (TSC) 1 (hamartin)/TSC2 (tuberin) complex is one of the targets of Akt for the modulation of mTORC1 activity. In the absence of Akt, the TSC1/TSC2 complex is a negative regulator of mTORC1. TSC2 functions as a GTPase-activating protein (GAP), converting a small G protein Ras homologue enriched in brain (Rheb-GTP) to the inactive GDP-bound form (Rheb-GDP) [141]. Once active, Rheb-GTP can directly interact with Raptor to activate mTORC1 and also regulate the binding of 4EBP1 to mTORC1 [142]. Akt phosphorylates TSC2 on multiple sites that leads to the destabilization of TSC2 and disruption of its interaction with TSC1. The phosphorylation of TSC2 on the residues of serine^939^, serine^981^, and threonine^1462^ can increase its binding to the anchor protein 14-3-3 and lead to the cellular sequestration by 14-3-3, disruption of the TSC1/TSC2 complex, and subsequent activation of Rheb and mTORC1 [143].

The proline rich Akt substrate 40 kDa (PRAS40) and I-kappaB kinase (IKK) also are targets of Akt to control the activation of mTORC1. PRAS40 can be phosphorylated on several residues including serine^183^, serine^212^, serine^221^, and threonine^246^[144,145]. The serine sites are targets of mTOR and the residue of threonine^246^ is the phosphorylation target of Akt. The phosphorylation of PRAS40 leads to its dissociation with Raptor [146] and promotes the binding of PRAS40 to the cytoplasmic docking protein 14-3-3 [147–149]. This removes PRAS40 from interacting with Raptor and facilitates the activation of mTORC1 [150]. Akt also has been shown to promote the activation of mTORC1 through IKKα. Loss of IKKα inhibits mTOR activation in Akt-active cells during inactivation of the negative PI 3-K regulator PTEN [151]. Within IKK, IKKα and IKKβ are catalytic subunits of IKK that possess serine/threonine kinase activity [152]. IKKα regulates mTOR activity by associating with Raptor that is Akt dependent [151]. In addition, IKKβ can phosphorylate TSC1 on serine^487^ and serine^511^ leading to the suppression of TSC1, disruption of TSC1/TSC2 complex, and the activation of mTORC1 [153]. Phosphorylation of IKKβ also has been associated with the activation of downstream pathways of mTOR signaling that involve p70S6K [154].

### 3.4. Apoptosis and Autophagy in the PI 3-K, Akt, and mTOR Cascade

The PI 3-K, Akt, and mTOR cascade closely govern cell survival during apoptosis and autophagy in the nervous system (Figure 1). PI 3-K and Akt activation can foster endothelial survival [128,155–161], limit neuronal injury [105,162–168], and block inflammatory cell death [59,81,106–108,169,170], and block neuronal injury [105,162–168]. A number of cellular pathways can be responsible for the activation of PI 3-K and Akt. For example, intracellular calcium release that is controlled by calmodulin activation leads to the association of calcium and calmodulin with the 85 kDa regulatory subunit of PI 3-K to activate Akt and promote neuronal survival [171,172]. Other pathways may be mediated through growth factors and cytokines, such as erythropoietin (EPO) [173]. For example, EPO induces the phosphorylation of Akt at serine^473^ to lead to its activation. EPO can protect dorsal root ganglion neurons in animal models of diabetes mellitus with streptozotocin through pathways that activate Akt [174]. EPO relies upon Akt activation in pathways that require sirtuins to maintain cerebral vascular cell survival during oxidative stress [159]. During EPO exposure, Akt is activated that results in the post-translational phosphorylation of forkhead transcription factors, such as FoxO proteins. Once phosphorylated, FoxO is sequestered in the cytoplasm by association with 14-3-3 proteins and transcription of “pro-apoptotic” genes is prevented [175]. Mammalian forkhead transcription factors of the O class (FoxO1, FoxO3, FoxO4, and FoxO6), such as FoxO3a, are intimately involved with cellular survival, metabolism, insulin sensitivity, and oxidative stress [176,177]. Akt limits apoptosis through the phosphorylation of FoxO proteins [176,178–181]. Akt phosphorylates the residue of serine^253^ of FoxO3a resulting in its export from the nucleus to the cytoplasm and blocking FoxO3a from activating apoptotic genes. Akt is required for EPO to promote protection such as during glyoxal-advanced glycation end products (AGEs) exposure [167], retinal detachment [182], β-amyloid (Aβ) exposure [80,183–185], hypoxia [156,186], and oxidative stress [187–189]. In addition, the downstream targets of Akt include BAD, caspase 9, and glycogen synthase kinase-3β (GSK-3β) [190–192]. Phosphorylation of GSK-3β, BAD, and caspase 9 by Akt prevents apoptosis and promotes cell longevity pathways [1,3,18,193]. Akt can phosphorylate GSK-3β at serine^9^ and inactivate this enzyme, this preventing GSK-3β from initiating an apoptotic pathway. Phosphorylation of Bad at serine^136^ by Akt can result in the inactivation of Bad and prevent neuronal apoptosis [159,194].

Similar to Akt, mTOR can rely upon pathways such as BAD to control apoptosis. mTOR also regulates apoptotic cell death through the mTOR signaling pathways of p70S6K and 4EBP1 [87,133,195]. Phosphorylation of BAD leads to the dissociation of BAD from Bcl-2/Bcl-x_L_ that prevents apoptotic cell death and increases BAD binding to the cytoplasmic docking protein 14-3-3. Activation of p70S6K also promotes the phosphorylation of BAD to limit apoptotic cell injury [195]. The activation of mTOR and p70S6K may also decrease apoptosis in non-neuronal astrocytes through pathways that can increase “anti-apoptotic” Bcl-2/Bcl-x_L_ expression [195]. Growth factors, such as insulin, prevent apoptosis in rat retinal neuronal cells against serum deprivation through activation of mTOR and p70S6K [196]. In addition, over-expression of wild type p70S6K increases cell protection by insulin. In contrast, over-expression of a dominant-negative mutant of p70S6K results in the loss of the ability of insulin to protect neurons [196]. Other growth factors similar to insulin, such as EPO [100,197], also have been reported to be dependent upon mTOR, p70S6K, and 4EBP1 for cytoprotection against apoptosis [145,184,198–200]. In regards to 4EBP1, inhibition of mTOR signaling by small interfering RNA (siRNA) inhibits phosphorylation of both p70S6K and 4EBP1 that results in apoptosis [201]. In the absence of mTOR activity, 4EBP1 has increased binding to eIF4E that can lead to the translation of apoptotic promoting proteins and also initiate autophagy [202].

mTOR also works in conjunction with Akt to offer cellular protection in the nervous system. mTOR and Akt are necessary for the protection of endothelial cells against apoptosis [203] and to prevent cell injury from FoxO3a [155,203]. Inflammatory cells succumb to apoptotic injury during oxidative stress if deprived of Akt and mTOR activation [184,204]. Administration of agents that enhance mTOR and Akt activity can prevent apoptotic dopaminergic neuronal death [205].

Akt also functions to modulate apoptosis through the mTOR signaling pathway of PRAS40. Phosphorylation of PRAS40 by Akt can block the activity of this substrate, lead to its dissociation from mTORC1 to allow mTOR activation, and prevent apoptotic cell injury [147,206,207]. PRAS40 is an important regulatory component for protection in the nervous system. Inhibition of PRAS40 activity can prevent cell death against oxidative stress and Aβ suggesting that PRAS40 may be a critical target to prevent neurodegeneration [147,206]. In addition, PRAS40 appears to be closely aligned with the apoptotic injury cascade since PRAS40 can directly control caspase 3 activation [147].

Under some circumstances such as during chronic disorders of neurodegeneration, inhibition of mTOR activity may be beneficial as opposed to activation of mTOR. Activation of mTOR and mTORC1 signaling can promote cell cycle induction that can be detrimental to post-mitotic neurons. Post-mitotic neurons that attempt to enter the cell cycle during AD do not replicate, but can result in apoptotic cell death [208,209]. In studies with amyloid oligomer exposure, neurons can be prevented from entering the cell cycle during the inhibition of mTOR and thus be protected from apoptosis [210]. Furthermore, blockade of the PI 3-K, Akt, and mTOR cascade can lead to the induction of autophagy that may be necessary for cell protection. Under some conditions that involve AD, PD, or HD, induction of autophagy can be cytoprotective. The degree of mTOR activation may be a significant variable in disorders that under some conditions can benefit from precise mTOR activity. During the early phases of autophagy, mTOR activity can be inhibited [211]. However, re-activation of mTOR appears to be required to continue with autophagy as long as elevated levels of mTOR activity do not lead to the eventual blockade of autophagy [212]. This modulation of mTOR with autophagy may be a conserved response in multiple cell systems that is governed by nutrient availability [212].

## 4. Clinical Disorders Modulated by PI 3-K, Akt, and mTOR

### 4.1 Diabetes Mellitus in the Nervous System

Throughout the world, more than 165 million individuals are afflicted with DM and it is expected that close to 400 million individuals by the year of 2030 will suffer from DM. The incidence of obesity in the population throughout the world is increasing at an alarming rate that ultimately leads to metabolic disease and diabetes mellitus (DM) [66]. Recent studies have shown that the duration of obese-years rather than body mass index (BMI) translates into a strong risk for developing DM [213]. Increased weight gain also leads to other disorders that may be a result of metabolic disease, such as coronary artery calcifications and the loss of cognition [68,214]. In younger individuals, impaired glucose tolerance is of significant concern, since those with impaired glucose tolerance have a greater than twice the risk for the development of diabetic complications than individuals with normal glucose tolerance [87,129].

**Figure 2:**
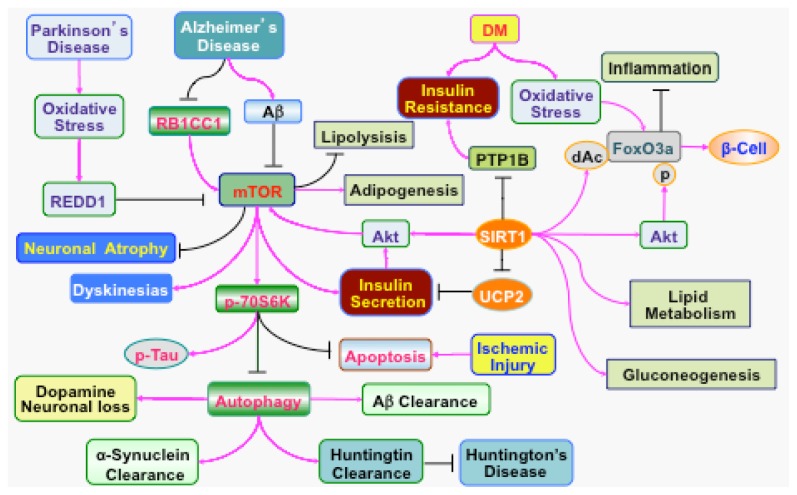
Modulation of neurodegenerative disorders through PI 3-K, Akt, mTOR, and associated pathways of SIRT1. Oxidative stress leads to cell injury in multiple neurodegenerative disorders. In Parkinson’s disease (PD), oxidative stress can lead to the induction of the stress response protein REDD1 that can inhibit the activation of mTOR. The accumulation of amyloid (Aβ) during Alzheimer’s disease (AD) also can block the activation of mTOR. In AD, retinoblastoma tumor suppressor (RB1) inducible Coiled-Coil 1 (RB1CC1), which functions to activate mTOR, is reduced, contributing to neuronal atrophy in AD. The activation of the downstream target of mTOR, p70 ribosome S6 kinase (p70S6K), by phosphorylation (p) prevents acute neuronal injury during stroke. However, inhibition of mTOR and p70S6K is required to promote autophagy and the clearance of aggregate prone proteins, such as α-synuclein, Aβ, and Huntingtin to prevent neuronal loss. A fine balance of mTOR activation is necessary in these disorders since mTOR can lead to dyskinesia in PD and activation of p70S6K has been associated with the promotion of the phosphorylation of tau protein contributing to formation of neurofibrillary tangles. During diabetes mellitus (DM), increasing oxidative stress results in insulin resistance, which can be ameliorated by SIRT1. Activation of SIRT1 can increase the secretion of insulin by repressing the mitochondrial uncoupling protein 2 (UCP2), promoting lipolysis, and increasing gluconeogenesis. SIRT1 also can increase insulin sensitivity by inhibiting tyrosine phosphatase 1B (PTP1B). Elevated levels of oxidative stress can reduce insulin sensitivity and enhance the activity of FoxO3a. FoxO3a has multiple roles that can interact with SIRT1 and influence β cell function as well as modulate inflammation. SIRT1 can increase FoxO3a activity through deacetylation (dAc). SIRT1 also can activate Akt that decreases the activity of FoxO3a through phosphorylation (p). Enhanced activity of mTOR increases insulin secretion, induces adipogenesis, and inhibits lipolysis that can influence nervous system complications of DM.

Given the progressive development of DM in the population, elucidation of novel pathways that can regulate the onset and progression of diabetic complications in the nervous system and related cardiovascular pathways would be highly desirable. One signaling pathway that is intimately tied to the PI 3-K, Akt, and mTOR pathway in DM involves sirtuins (Figure 2). Sirtuins are class III NAD^+^-dependent protein histone deacetylases that are the mammalian homologues of Sir2 of the yeast silent information regulator-2 (Sir2) [1]. Of the seven mammalian homologues of Sir2, SIRT1 has been shown to be protective against cellular injury during DM. SIRT1 activation prevents endothelial senescence during hyperglycemia, blocks atherosclerotic lesions during elevated lipid states, modulates adipocyte differentiation, and prevents endothelial cell apoptosis during experimental diabetes [1,128,159,215,216]. SIRT1 also can increase lifespan in higher organisms such as Drosophila and protect cells from oxidative stress [102,217]. Loss of SIRT1 is associated with insulin resistance. Gene deletion or inhibition of SIRT1 impairs insulin signaling by interfering with insulin stimulated insulin receptor phosphorylation and glycogen synthase [218]. In contrast, over-expression of SIRT1 decreases hepatic steatosis and improves insulin sensitivity that leads to improved glucose homeostasis [219]. SIRT1 can increase insulin signaling in insulin-sensitive organs through Akt and PI 3-K [220] and can stimulate glucose-dependent insulin secretion from pancreatic β cells by repressing the uncoupling protein (UCP) gene UCP2 [221]. SIRT1 also controls insulin sensitivity through the inhibition of tyrosine phosphatase1B (PTP1B). SIRT1 over-expression or SIRT1 activation can reduce both PTP1B mRNA and protein levels during insulin-resistance. However, an increase in PTP1B expression prevents SIRT1 mediated glucose uptake and insulin receptor phosphorylation in response to insulin stimulation [218]. SIRT1 also may improve insulin sensitivity through the regulation of fat mobilization, gluconeogenesis, and inflammation [1,3,215].

Agents such as EPO increase endogenous SIRT1 activity and foster nuclear subcellular trafficking of SIRT1 [159]. SIRT1 increases Akt activity that is considered to be a principle pathway for cellular proliferation, survival, and for mediating insulin signaling [6,52,55,128,159,222]. SIRT1 activates Akt1 to also control the phosphorylation and subcellular trafficking of FoxO3a [159]. SIRT1 relies upon the Akt pathway for cytoprotection and the subsequent phosphorylation of target genes such as FoxOs [159,220]. However, SIRT1 may control a fine balance over FoxO activity since acetylation of FoxOs also can limit their transcriptional activity. SIRT1 can lead to the deacetylation of FoxOs to increase the activity of these transcription factors [178,181]. Yet, the deacetylation of FoxOs by SIRT1 may ultimately lead to degradation of these transcription factors [223]. SIRT1 also may rely upon the regulation of FoxO transcription factors to protect against inflammation, to promote β-cell function during DM, and to maintain endothelial survival during oxidative stress [3,181].

Signaling through SIRT1, PI 3-K, and Akt during cell metabolism also is dependent upon mTOR. For example, loss of SIRT1 expression leads to hepatic glucose overproduction, hyperglycemia, products of oxidative stress, and inhibition of the gene encoding Rictor that results in impaired TORC2 and Akt signaling [224]. Under some conditions, SIRT1 and mTOR may have an inverse relationship. SIRT1 can attenuate hepatic steatosis, ameliorate insulin resistance, and restore glucose homeostasis primarily through the inhibition of mTORC1 [219]. SIRT1 also may promote neuronal growth through pathways that inhibit mTOR signaling [225].

Independently, mTOR signaling also may be necessary for maintenance of insulin function. Activation of p70S6K, and inhibition of 4EBP1 in pancreatic β-cells in murine models of DM, results in improved insulin secretion and resistance to β-cell streptozotocin toxicity and obesity [226]. However, loss of p70S6K activity with combined loss of the mTORC2 substrate Akt2 results in defects in both insulin action and β-cell function, suggesting that both mTOR components are required to maintain insulin signaling and prevent DM [227]. Furthermore, mTOR inhibition with rapamycin application leads to insulin resistance, reduces β-cell function and mass, limits insulin secretion, and results in DM [228]. Loss of mTOR signaling with inhibition of p70S6K also can result in hypoinsulinemia, glucose intolerance, insulin insensitivity to glucose secretion, and a decrease in pancreatic β-cell size [25,87]. Although inhibition of mTOR can reduce food intake and prevent fat-diet induced obesity in mice, loss of mTOR activity also attenuates glucose uptake and metabolism in skeletal muscle through the prevention of insulin generated Akt activation and alteration in the translocation of glucose transporters to the plasma membrane [127].

mTOR activity also can regulate lipid metabolism. Activation of mTOR may be essential for adipogenesis and adipocyte differentiation [229,230]. Application of rapamycin has been shown to significantly reduce expression of most adipocyte markers including peroxisome proliferators-activated receptor-γ (PPAR-γ), adipsin, adipocyte protein 2 (aP2), α-adducin (ADD1)/sterol regulatory element-binding protein 1(SREBP1c), and FAS [229]. Rapamycin also prevents intracellular lipid accumulation in 3T3-L1 and 3T3-F442A cells [229]. In addition, activation of mTOR inhibits lipolysis. For example, activation of mTORC1 signaling in 3T3-L1 adipocytes by ectopic expression of Rheb inhibits adipose triglyceride lipase and hormone-sensitive lipase and promotes intracellular accumulation of triglycerides. In contrast, inhibition of mTORC1 signaling by rapamycin or by knockdown of Raptor results in an increase in the expression of lipase and lipolysis [231] and leads to weight loss in gerbil, rats, and humans [228,232]. The ability of mTOR to regulate lipid metabolism has been associated with lipin, a magnesium-dependent phosphatidic acid phosphatase. Lipin plays a role in the regulation of fat cell differentiation and triglyceride synthesis [233]. Mutations in the *Lpin1* gene result in hepatic steatosis in fld mice, a genetic model of lipodystrophy [234]. Lipin may function downstream of the peroxisome proliferator-activated receptor γ (PPARγ) coactivator 1alpha (PGC-1α) and selectively activates a PGC-1α mediated fatty acid oxidation and mitochondrial oxidative phosphorylation, suppresses lipogenesis, and lowers circulating lipid levels [87,235]. Activation of mTOR can increase the phosphorylation of lipin and mediate insulin induced lipin phosphorylation, resulting in the release of lipin from intracellular membranes [236].

In the central nervous system, mTOR also has a role in regulating food intake in the hypothalamus. mTOR is expressed ubiquitously in the nervous system. Phosphorylated mTOR and P70S6K has been shown to be expressed in the hippocampus, thalamus, cortex, and paraventricular (PVN) and arcuate (ARC) nuclei of the hypothalamus [237]. The ARC nuclei contain at least two populations of neurons, including orexigenic neurons that express both neuropeptide Y (NPY) and agouti-related peptide (AgRP) and anorexigenic neurons that express proopiomelanocortin (POMC) and cocaine- and amphetamine-regulated transcript (CART). Each of these agents is linked to the regulation of cellular energy homeostasis. mTOR and p70S6K exist in up to 90% of ARC NPY/AgRP neurons and in approximately 45% of ARC POMC/CART neurons. Central administration of leucine increases hypothalamic mTOR signaling and decreases food intake and body weight. The hormone leptin increases hypothalamic mTOR activity and inhibition of mTOR signaling has been associated with anorexia [237]. Leptin significantly results in the phosphorylation of p70S6K and p90 ribosomal S6 kinase 1 (RSK1) in mice placed on a low-fat diet and leads to hyperphagia, weight gain, and leptin resistance during diet-induced obesity [238]. Nutrient availability and insulin regulate leptin expression. Yet, mTORC1 also plays a role in leptin expression in adipose cells, since up-regulation of mTORC1 in 3T3-L1 adipocytes via stable expression of either constitutively active Rheb or dominant-negative AMP activated protein kinase (AMPK) results in a significant increase in leptin expression [239]. AMPK can phosphorylate tuberin (TSC2) and inhibit mTORC1 [240].

### 4.2. Acute Injury in the Nervous System

The PI 3-K, Akt, and mTOR pathways play a significant role during acute injury in the nervous system (Figure 2). For example, EPO activates the PI 3-K and Akt pathways to protect neurons, vascular cells, and immune cells during oxidative stress [80,155,156,159,167,169,174,175,182,183,187,200,241,242]. Agents that can increase the expression of Raptor are associated with neuroprotection during hypoxia in invertebrate models of stroke [243]. Furthermore, agents that increase activity of Akt, mTOR, and p70S6K also can reduce cerebral infarct size [244]. Activation of mTOR is necessary in primary cerebral microglia [80,184,204,206] and in neurons [25,147] to prevent apoptotic cell death during oxygen-glucose deprivation. Following acute spinal cord injury, an increase in mTOR expression and p70S6K activity also may be required for functional improvement [245]. Studies with bisperoxovanadium that can enhance the activities of Akt and mTOR have been demonstrated to reduce motor neuron death, increase tissue sparing, and reduce cavity formation after spinal cord injury in rats [246]. ATP administration that increases Akt, mTOR, and p70S6K signaling is accompanied by improved locomotor function following spinal cord injury [247]. mTOR in conjunction with other pathways such as signal transducers and activators of transcription (STAT) pathways can foster axonal regeneration [248]. For example, axonal regeneration is increased in adult retinal ganglion cells and in corticospinal neurons following injury paradigms with mTOR activation [249,250].

However, some conditions may require mTOR blockade to promote neuronal protection and autophagy in conjunction with increased activity of the PI 3-K and Akt axis [111]. Inhibition of mTOR and p70S6K activities also improves functional recovery in closed head injury models [251]. Blockade of mTOR has been shown to promote autophagy, inhibit mTOR-mediated inflammation, reduce neural tissue damage, and limit locomotor impairment following spinal cord injury [252]. Inhibition of PTEN (phosphatase and tensin homolog deleted on chromosome 10) that results in enhanced mTOR activity results in increased cerebral infarction [253]. Inhibition of mTOR signaling also prevents cerebral vasospasm and preserves endothelial cell function in animal models of subarachnoid hemorrhage [254].

During disorders of epilepsy that can be a recurrent acute disability, mTOR inhibition may be beneficial. Aberrant or significant mTOR activity is believed to interfere with normal brain function and lead to epilepsy. Inhibition of mTOR activity during kainate-induced epilepsy decreases neuronal cell death, neurogenesis, mossy fiber sprouting, and the development of spontaneous epilepsy [255]. Chronic hippocampal infusion of rapamycin that blocks mTOR signaling also limits mossy fiber sprouting in rat pilocarpine models of temporal lobe epilepsy [256]. mTOR signaling also is considered to be one mechanism for seizure disorders that occur in tuberous sclerosis (TS) [257]. Mutations of TSC1 and TSC2 that lead to hyperactive mTOR result in a high incidence of epilepsy [258]. Early inhibition of mTOR signaling in animal models of TS can prevent astrogliosis and neuronal dysfunction [259].

### 4.3. Chronic Neurodegeneration

Similar to disorders with acute nervous system injury, the temporal course and level of PI 3-K, Akt, and mTOR activation during chronic neurodegenerative disorders that can progress during aging can influence cellular survival and clinical outcome (Figure 2). During disorders such as AD, a minimum level of the PI 3-K, Akt, and mTOR pathway may be required. Since Aβ is toxic to cells [184,260], activation of the PI 3-K and Akt pathways has been shown to prevent Aβ toxicity [103,168,184,261–263]. In regards to mTOR, blockade of mTOR activity may lead to neuronal atrophy in AD. Insufficiency of retinoblastoma tumor suppressor (RB1) inducible Coiled-Coil 1 (RB1CC1) has been observed in the brains of AD patients. In these patients, RB1CC1 appears to be necessary for neurite growth and to maintain mTOR signaling, but the reduced expression of RB1CC1 leads to reduced mTOR activity, neuronal apoptosis, and neuronal atrophy [264]. A decrease in mTOR activity in peripheral lymphocytes also appears to correlate with the progression of AD [265] and inhibition of mTOR activity has been shown to impair memory consolidation [266]. Loss of mTOR signaling also has been shown to impair long-term potentiation and synaptic plasticity in models of AD [267]. In addition, Aβ can block the activation of mTOR and p70S6K in neuroblastoma cells and in lymphocytes of patients with AD [268]. Activation of mTOR and p70S6K has been shown to prevent cell death during Aβ exposure in microglia, cells that are necessary for the removal of Aβ [184].

Additional studies provide further support for the premise that the degree of activity for the PI 3-K, Akt, and mTOR pathways may be an important factor for the treatment of neurodegenerative disorders, such as AD. In fact, some investigations suggest that inhibition of PI 3-K, Akt, and mTOR signaling may be necessary to achieve therapeutic benefit. For example, an increase in the phosphorylated level of Akt substrates, such as mTOR, GSK-3β, and tau protein have been observed in AD, suggesting that these substrates may promote AD progression [269]. Hyper-activation of PI 3-K and Akt associated with decreased calmodulin degradation in lymphoblasts from patients with AD also has been suggested as a potential detriment to cell survival [270]. p70S6K activation also has been associated with hyperphosphorylated tau formation and potential neurofibrillary accumulation in AD patients [271]. In addition, mTOR inhibition that can lead to autophagy in murine models of AD has been shown to improve memory and limit Aβ levels [93].

Inhibition of the PI 3-K, Akt, and mTOR pathway also may be necessary for the treatment of HD, an autosomal dominant disorder characterized by the degeneration of striatal GABAergic projecting neurons that result in involuntary movements and cognitive impairment. Activation of autophagy and the inhibition of mTOR are considered vital for the clearing of aggregate-prone proteins in disorders such as HD [272]. HD is the result of neuronal intracellular aggregates of huntingtin protein mutations that produce abnormally expanded polyglutamine in the N-terminal region of the *huntingtin* gene and lead to neuronal cell death. As a result, inhibition of mTOR signaling that can promote autophagy may represent a potential therapeutic strategy for HD. Blockade of mTOR activity has been demonstrated to enhance autophagic clearance of proteins with long polyglutamines and a polyalanine-expanded protein [273], attenuate huntingtin accumulation and cell death in cell models of HD, and protect against neurodegeneration in a fly model of HD [274]. Small molecular enhancers of rapamycin also have been shown to promote autophagy with both mTOR dependent and independent mechanisms to increase the clearance of a mutant huntingtin fragment in HD cell models [275]. The rapamycin analog CCI-779 also improves behavioral performance and decreases aggregate formation in a mouse model of HD [274]. Yet, some experimental models of HD suggest that inhibition of only mTORC1 may be insufficient to alter autophagy or huntingtin accumulation. The combined inhibition of mTORC1 and mTORC2 is required for autophagy and reductions in huntingtin accumulation, suggesting that multiple components of the mTOR pathway may modulate cell survival in HD [276]. Other studies support this premise to demonstrate that decreased activity of p70S6K protects against early decline in motor performance with beneficial effects on muscle function, but mutant huntingtin levels in the brain are not affected [277]. Prevention of neuronal demise by mTOR may work in concert with growth arrest and DNA damage protein 34 (GADD34). GADD34 leads to the dephosphorylation of TSC2 and induction of autophagy in cell models of HD with increased cell survival during GADD34 over-expression [27].

In PD, a movement disorder characterized by resting tremor, rigidity, and bradykinesia as a result of the loss of dopaminergic neurons in the substantia nigra, mTOR inactivation and promotion of autophagy may preserve dopaminergic neurons. During inhibition of mTOR and the activation of autophagy, the accumulation of toxic α-synuclein in transgenic mice is reduced and neurodegeneration is decreased in models of PD [278]. Yet, only portions of the PI 3-K, Akt, and mTOR pathway may be necessary for the treatment of PD. Treatment with rapamycin protects neurons that has been shown to be dependent upon Akt activation, but not associated with mTOR signaling [166]. In addition, activation of 4EBP1 during mTOR inhibition can suppress pathologic experimental phenotypes of PD including degeneration of dopaminergic neurons in Drosophila [279].

Other studies indicate that at least some activity of the mTOR pathway is required for neuronal protection in PD. Decreased mTOR activity may lead to neuronal injury in PD through pathways such as autophagy. Inhibition of mTOR signaling during oxidative stress leads to cell death in dopaminergic neurons by enhancing autophagic neuronal death [205]. The stress response protein REDD1 (RTP801), an inhibitor of mTORC1 activity [280], also is up-regulated in dopaminergic neurons in PD patients [281] and can inactivate Akt and mTOR. REDD1 is highly expressed in several cellular models of PD such as treatment with 6–hydroxydopamine (6-OHDA), MPTP, and rotenone [281]. REDD1 is regarded as a potential contributor to neuronal degeneration in PD, since gene silencing of REDD1 is neuroprotective against 6-OHDA [166]. Loss of mTOR activity and the chronic activation of the mTOR pathway 4EBP1 by leucine-rich repeat kinase 2 (LRRK2), a site for dominant mutations PD, is believed to alter protein translation and lead to the loss of dopaminergic neurons [282]. Interestingly, elevated levels of activity in the PI 3-K, Akt, and mTOR pathways can be detrimental in PD. Treatment with derivatives a dopamine, such as L-DOPA, lead to dopamine D1 receptor-mediated activation of mTORC1 resulting in dyskinesia [283].

## 5. Conclusions and Perspectives

Oxidative stress plays a significant role in the pathology of numerous disorders of the nervous system whether they are of acute or chronic origin during aging. Intimately tied to oxidative stress and the pathways that can determine cell survival are PI 3-K, Akt, and mTOR. The PI 3-K, Akt, and mTOR cascade is involved in the modulation of apoptosis, autophagy, disorders of cellular metabolism, acute nervous system injury, and chronic neurodegeneration. Targeting these pathways offers new inroads for the development of novel therapies that can either prevent or reverse the progression of diabetic complications in the nervous system and nervous system dysfunction. New studies suggest that consideration should be given for the modulation of individual components of the PI 3-K, Akt, and mTOR pathway, since neuronal protection in some cases such as those involving PD may be dependent upon one component such as Akt, but not other downstream components such as mTOR [166]. New work is focusing upon agents that can target different Akt classes with the alkyl-lysophospholipids and small molecule inhibitors of Akt. Alternate strategies that consider PDK1 and eIF4E are also under study. In other scenarios, such as with HD, broader aspects of the cascade may be required that can foster the reduction of toxic cellular aggregates [276]. In preclinical studies, modulation of the PI 3-K, Akt, and mTOR cascade can increase radiosensitivity against tumor cell growth and the vascular supply of tumors [284]. In addition, it appears that mTOR signaling components are required to maintain insulin signaling and prevent DM, since loss of p70S6K activity with the combined loss of the mTORC2 substrate Akt2 results in defects in both insulin action and β-cell function [227]. One must also take into account additional mechanisms that are tied to the PI 3-K, Akt, and mTOR pathways to promote neuroprotection and cellular energy homeostasis, such as GADD34 [27], forkhead transcription factors [110,177,178,285], Raf-mitogen activated protein kinases [286], and SIRT1 [1,3,215].

However, modulation of the PI 3-K, Akt, and mTOR cascade, especially during times of activation is not without its concerns. PI 3-K, Akt, and mTOR are cellular proliferative pathways that can promote aggressive tumor growth. As a result, the United States Food and Drug Administration has approved several rapamycin (sirolimus) and rapamycin derivative compounds (“rapalogs”) for the treatment of nervous system cancers that include subependymal giant cell astrocytoma associated with tuberous sclerosis (everolimus) and neuroendocrine pancreatic tumors (everolimus) [73,99]. Early clinical trials using everolimus for the treatment of advanced neuroendocrine tumors suggest that progression free survival can be improved [287]. Prevention of PI 3-K and Akt activation also can block medulloblastoma growth [288].

In addition to these considerations, parameters related to the temporal administration of agents that can modulate the PI 3-K, Akt, and mTOR signaling and the pathways of cell death that should be targeted appear to be critical. For example, recent phase III trials in relation to AD suggest that attempts to reduce Aβ toxicity in the brains of patients with AD through immunotherapy with Bapineuzumab or with Solanezumab (LY2062430) may have had limited clinical success as a result of not instituting therapy prior to significant clinical disease presentation or progression. In animal models of TS, early inhibition of mTOR signaling rather than late treatment has been shown to prevent astrogliosis, premature death, and seizures [259]. Early rather than late mTOR inhibition also can reduce plaques, tangles, and loss of cognition in murine models of AD [289]. Additional work suggests that the duration of activation of PI 3-K, Akt, and mTOR may be a significant factor for influencing cell survival. Inhibition of mTOR can impair long-term potentiation and synaptic plasticity in models of AD [267] and activation of mTOR signaling can block inflammatory cell death during Aβ exposure [184]. Yet, excessive activation of mTOR can lead to dyskinesia patients with PD [283].

In regards to cell death pathways, apoptosis and autophagy appear to have a complex relationship. During toxin exposure that involves methamphetamine, cell death proceeds through apoptosis and autophagy by inhibiting the disassociation of the Bcl-2/Beclin 1 complex [89], an anti-apoptotic protein that blocks autophagy through its inhibitory interaction with Beclin 1 [290]. Autophagy and apoptosis also can have opposing roles. Some studies report that progression of apoptosis may require the inhibition of autophagy [75,90,291]. In relation to nervous system disorders, it remains unclear when pathways such as autophagy may be beneficial. During oxidative stress, autophagy can lead to cell death in cerebral astrocytes [82], in cortical neurons [83], and in spinal cord motor neurons [84]. Activation of the PI 3-K, Akt, mTOR pathway that can block autophagy may be necessary to protect against AGEs and complications of DM with atherosclerosis [97], prevent spinal cord injury [247], and maintain synaptic plasticity [267]. However, autophagy may be necessary during inhibition of mTOR signaling to improve cognitive function, limit Aβ toxicity [93], and clear mutant huntingtin in HD [275]. Over the next several years, further studies that can continue to unravel the cellular pathways governed by the PI 3-K, Akt, and mTOR cascade and the significant biological role this cascade holds in specific disorders of the nervous system will offer the greatest potential to target these pathways for robust treatments against neurodegenerative disorders.
